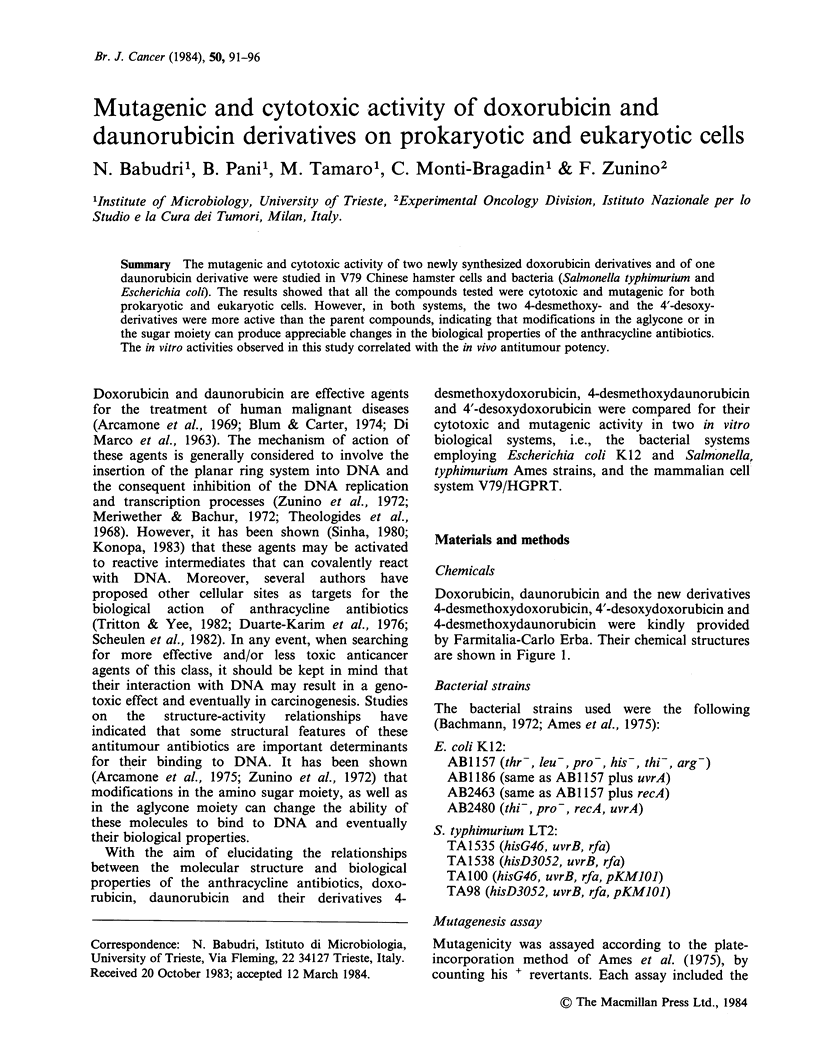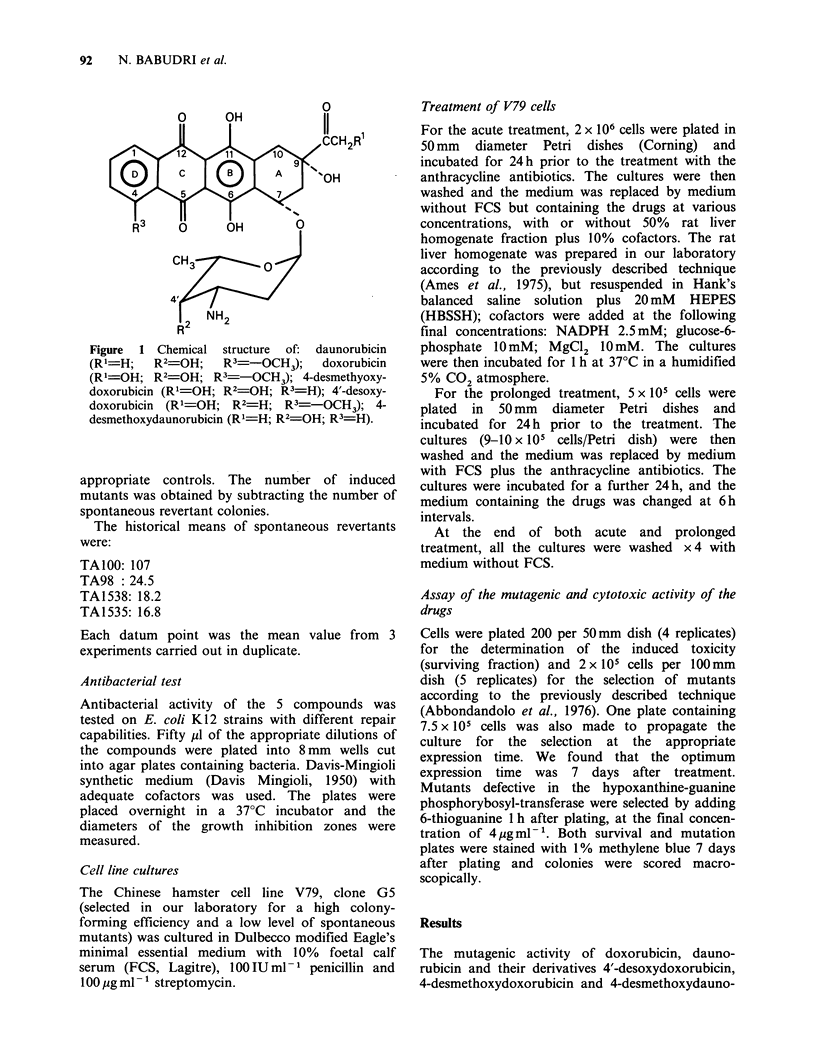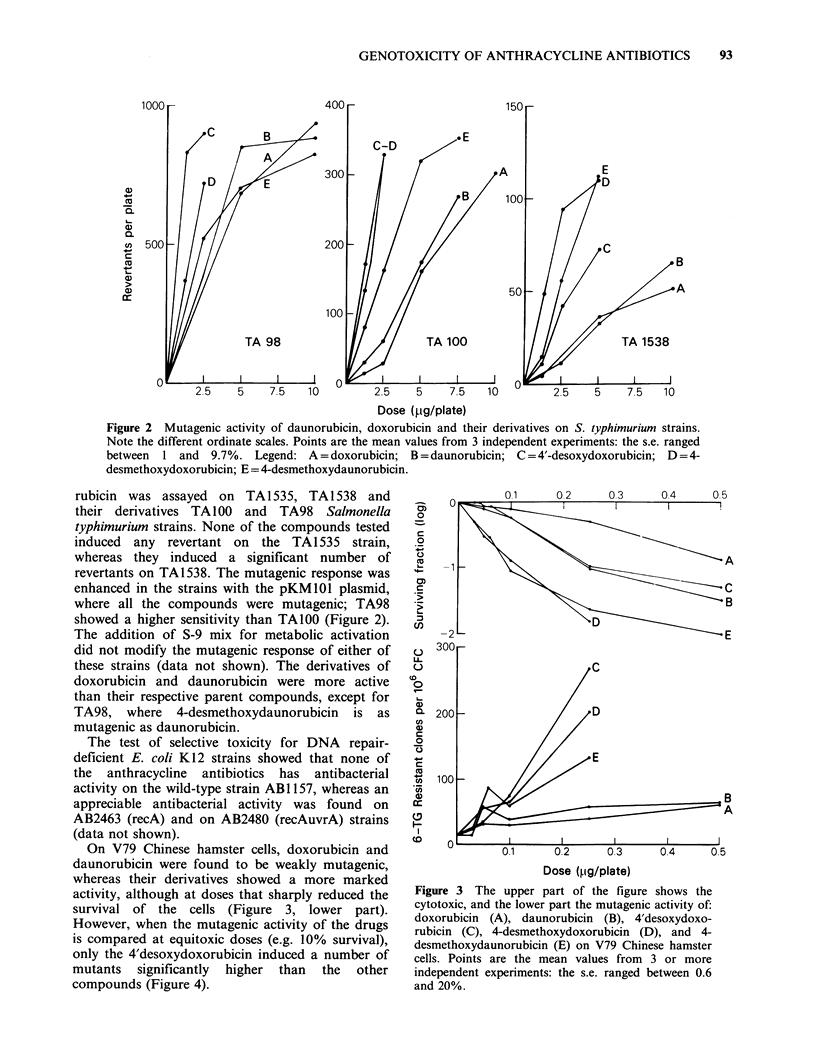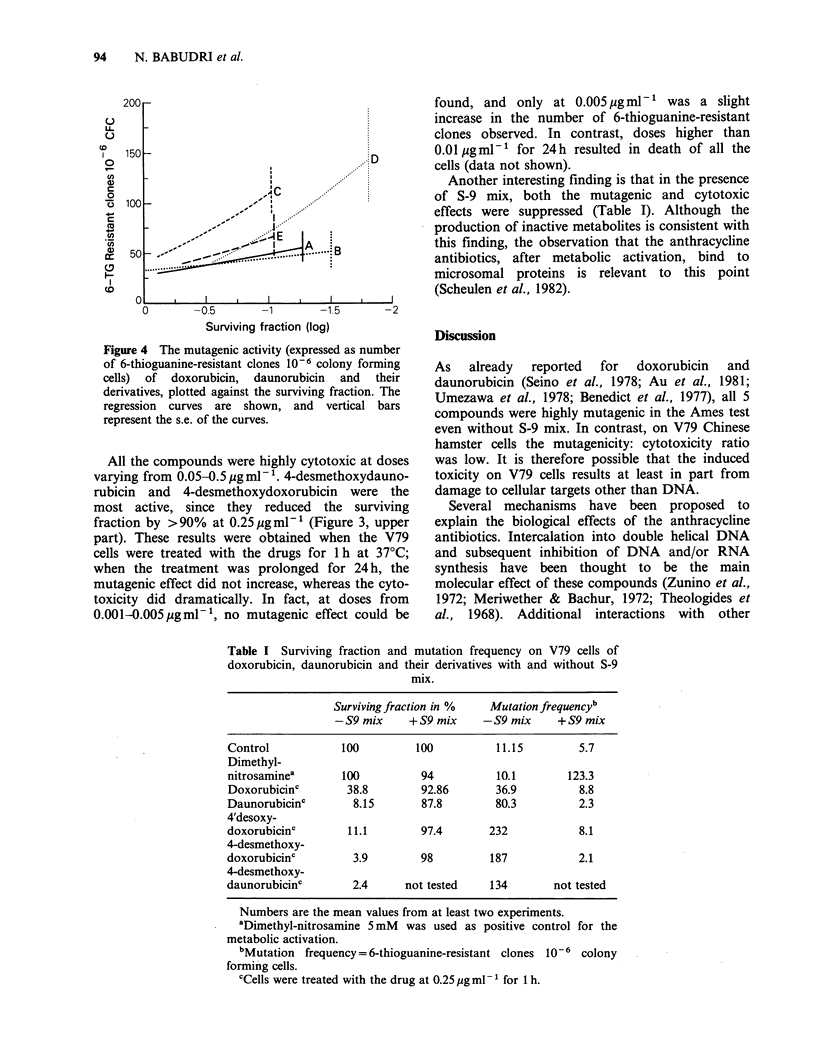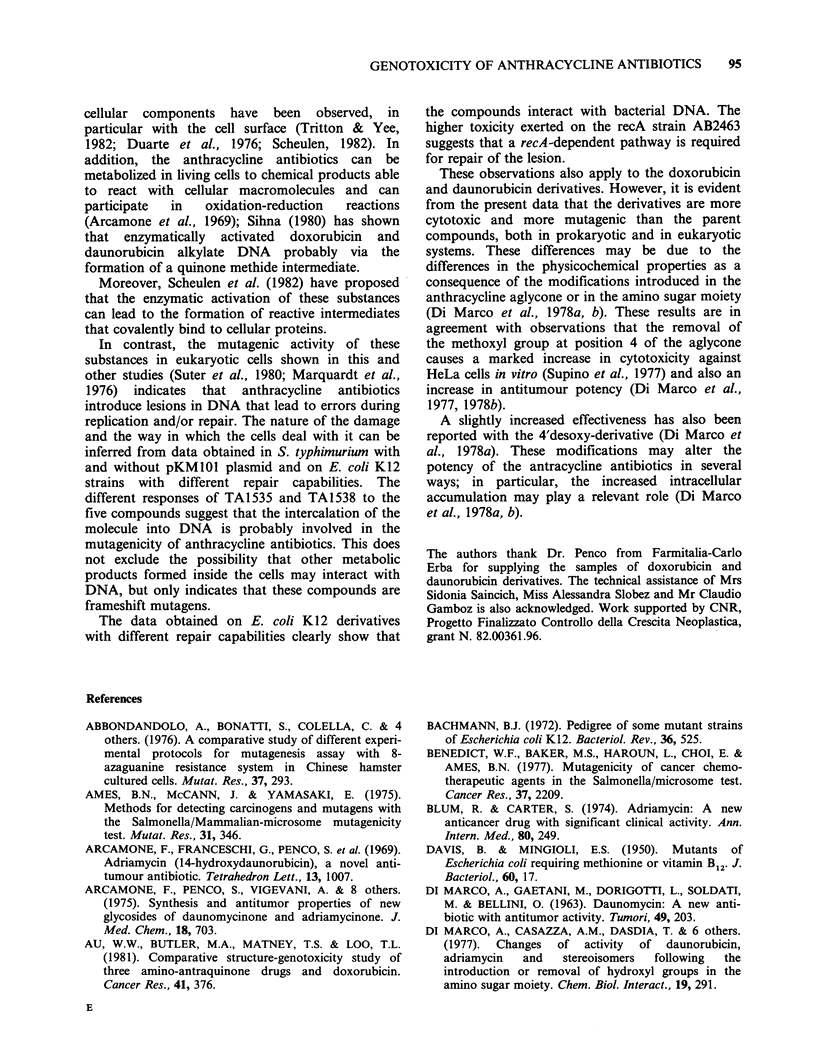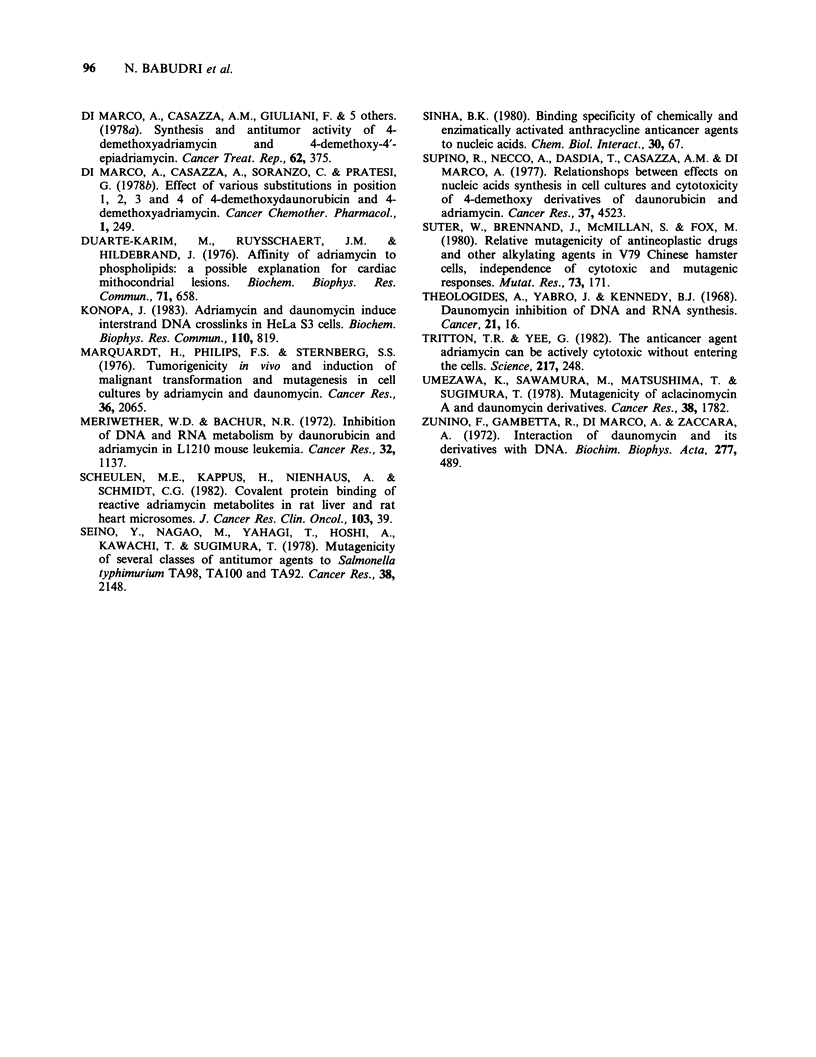# Mutagenic and cytotoxic activity of doxorubicin and daunorubicin derivatives on prokaryotic and eukaryotic cells.

**DOI:** 10.1038/bjc.1984.143

**Published:** 1984-07

**Authors:** N. Babudri, B. Pani, M. Tamaro, C. Monti-Bragadin, F. Zunino

## Abstract

The mutagenic and cytotoxic activity of two newly synthesized doxorubicin derivatives and of one daunorubicin derivative were studied in V79 Chinese hamster cells and bacteria (Salmonella typhimurium and Escherichia coli). The results showed that all the compounds tested were cytotoxic and mutagenic for both prokaryotic and eukaryotic cells. However, in both systems, the two 4-desmethoxy- and the 4'-desoxy-derivatives were more active than the parent compounds, indicating that modifications in the aglycone or in the sugar moiety can produce appreciable changes in the biological properties of the anthracycline antibiotics. The in vitro activities observed in this study correlated with the in vivo antitumour potency.


*Br. J. Cancer* (1984), **50**, 91–96

### Mutagenic and cytotoxic activity of doxorubicin and daunorubicin derivatives on prokaryotic and eukaryotic cells

N. Babudri[1], B. Pani[1], M. Tamaro[1], C. Monti-Bragadin[1] & F. Zunino[2]

[1]*Institute of Microbiology, University of Trieste,* [2]*Experimental Oncology Division, Istituto Nazionale per lo Studio e la Cura dei Tumori, Milan, Italy.*

**Summary** The mutagenic and cytotoxic activity of two newly synthesized doxorubicin derivatives and of one daunorubicin derivative were studied in V79 Chinese hamster cells and bacteria (*Salmonella typhimurium* and *Escherichia coli*). The results showed that all the compounds tested were cytotoxic and mutagenic for both prokaryotic and eukaryotic cells. However, in both systems, the two 4-desmethoxy- and the 4′-desoxy-derivatives were more active than the parent compounds, indicating that modifications in the aglycone or in the sugar moiety can produce appreciable changes in the biological properties of the anthracycline antibiotics. The *in vitro* activities observed in this study correlated with the *in vivo* antitumour potency.

Doxorubicin and daunorubicin are effective agents for the treatment of human malignant diseases (Arcamone *et al.*, 1969; Blum & Carter, 1974; Di Marco *et al.*, 1963). The mechanism of action of these agents is generally considered to involve the insertion of the planar ring system into DNA and the consequent inhibition of the DNA replication and transcription processes (Zunino *et al.*, 1972; Meriwether & Bachur, 1972; Theologides *et al.*, 1968). However, it has been shown (Sinha, 1980; Konopa, 1983) that these agents may be activated to reactive intermediates that can covalently react with DNA. Moreover, several authors have proposed other cellular sites as targets for the biological action of anthracycline antibiotics (Tritton & Yee, 1982; Duarte-Karim *et al.*, 1976; Scheulen *et al.*, 1982). In any event, when searching for more effective and/or less toxic anticancer agents of this class, it should be kept in mind that their interaction with DNA may result in a genotoxic effect and eventually in carcinogenesis. Studies on the structure-activity relationships have indicated that some structural features of these antitumour antibiotics are important determinants for their binding to DNA. It has been shown (Arcamone *et al.*, 1975; Zunino *et al.*, 1972) that modifications in the amino sugar moiety, as well as in the aglycone moiety can change the ability of these molecules to bind to DNA and eventually their biological properties.

With the aim of elucidating the relationships between the molecular structure and biological properties of the anthracycline antibiotics, doxorubicin, daunorubicin and their derivatives 4-desmethoxydoxorubicin, 4-desmethoxydaunorubicin and 4′-desoxydoxorubicin were compared for their cytotoxic and mutagenic activity in two *in vitro* biological systems, i.e., the bacterial systems employing *Escherichia coli* K12 and *Salmonella typhimurium* Ames strains, and the mammalian cell system V79/HGPRT.

Correspondence: N. Babudri, Istituto di Microbiologia, University of Trieste, Via Fleming, 22 34127 Trieste, Italy.
Received 20 October 1983; accepted 12 March 1984.

### Materials and methods

### *Chemicals*

Doxorubicin, daunorubicin and the new derivatives 4-desmethoxydoxorubicin, 4′-desoxydoxorubicin and 4-desmethoxydaunorubicin were kindly provided by Farmitalia-Carlo Erba. Their chemical structures are shown in Figure 1.

### *Bacterial strains*

The bacterial strains used were the following (Bachmann, 1972; Ames *et al.*, 1975):

*E. coli* K12:
- AB1157 (*thr*⁻, *leu*⁻, *pro*⁻, *his*⁻, *thi*⁻, *arg*⁻)
- AB1186 (same as AB1157 plus *uvrA*)
- AB2463 (same as AB1157 plus *recA*)
- AB2480 (*thi*⁻, *pro*⁻, *recA*, *uvrA*)

*S. typhimurium* LT2:
- TA1535 (*hisG46, uvrB, rfa*)
- TA1538 (*hisD3052, uvrB, rfa*)
- TA100 (*hisG46, uvrB, rfa, pKM101*)
- TA98 (*hisD3052, uvrB, rfa, pKM101*)

### *Mutagenesis assay*

Mutagenicity was assayed according to the plate-incorporation method of Ames *et al.* (1975), by counting his $^+$ revertants. Each assay included the

© The Macmillan Press Ltd., 1984

**Figure 1** Chemical structure of: daunorubicin ($R^1$=H; $R^2$=OH; $R^3$=—$OCH_3$); doxorubicin ($R^1$=OH; $R^2$=OH; $R^3$=—$OCH_3$); 4-desmethyoxy-doxorubicin ($R^1$=OH; $R^2$=OH; $R^3$=H); 4′-desoxy-doxorubicin ($R^1$=OH; $R^2$=H; $R^3$=—$OCH_3$); 4-desmethoxydaunorubicin ($R^1$=H; $R^2$=OH; $R^3$=H).

appropriate controls. The number of induced mutants was obtained by subtracting the number of spontaneous revertant colonies.

The historical means of spontaneous revertants were:

TA100: 107
TA98 : 24.5
TA1538: 18.2
TA1535: 16.8

Each datum point was the mean value from 3 experiments carried out in duplicate.

*Antibacterial test*

Antibacterial activity of the 5 compounds was tested on *E. coli* K12 strains with different repair capabilities. Fifty $\mu$l of the appropriate dilutions of the compounds were plated into 8 mm wells cut into agar plates containing bacteria. Davis-Mingioli synthetic medium (Davis Mingioli, 1950) with adequate cofactors was used. The plates were placed overnight in a 37°C incubator and the diameters of the growth inhibition zones were measured.

*Cell line cultures*

The Chinese hamster cell line V79, clone G5 (selected in our laboratory for a high colony-forming efficiency and a low level of spontaneous mutants) was cultured in Dulbecco modified Eagle's minimal essential medium with 10% foetal calf serum (FCS, Lagitre), 100 IU $ml^{-1}$ penicillin and 100 $\mu g\ ml^{-1}$ streptomycin.

*Treatment of V79 cells*

For the acute treatment, $2 \times 10^6$ cells were plated in 50 mm diameter Petri dishes (Corning) and incubated for 24 h prior to the treatment with the anthracycline antibiotics. The cultures were then washed and the medium was replaced by medium without FCS but containing the drugs at various concentrations, with or without 50% rat liver homogenate fraction plus 10% cofactors. The rat liver homogenate was prepared in our laboratory according to the previously described technique (Ames *et al.*, 1975), but resuspended in Hank's balanced saline solution plus 20 mM HEPES (HBSSH); cofactors were added at the following final concentrations: NADPH 2.5 mM; glucose-6-phosphate 10 mM; $MgCl_2$ 10 mM. The cultures were then incubated for 1 h at 37°C in a humidified 5% $CO_2$ atmosphere.

For the prolonged treatment, $5 \times 10^5$ cells were plated in 50 mm diameter Petri dishes and incubated for 24 h prior to the treatment. The cultures (9–10 $\times 10^5$ cells/Petri dish) were then washed and the medium was replaced by medium with FCS plus the anthracycline antibiotics. The cultures were incubated for a further 24 h, and the medium containing the drugs was changed at 6 h intervals.

At the end of both acute and prolonged treatment, all the cultures were washed $\times 4$ with medium without FCS.

*Assay of the mutagenic and cytotoxic activity of the drugs*

Cells were plated 200 per 50 mm dish (4 replicates) for the determination of the induced toxicity (surviving fraction) and $2 \times 10^5$ cells per 100 mm dish (5 replicates) for the selection of mutants according to the previously described technique (Abbondandolo *et al.*, 1976). One plate containing $7.5 \times 10^5$ cells was also made to propagate the culture for the selection at the appropriate expression time. We found that the optimum expression time was 7 days after treatment. Mutants defective in the hypoxanthine-guanine phosphorybosyl-transferase were selected by adding 6-thioguanine 1 h after plating, at the final concentration of 4 $\mu g\ ml^{-1}$. Both survival and mutation plates were stained with 1% methylene blue 7 days after plating and colonies were scored macroscopically.

### Results

The mutagenic activity of doxorubicin, daunorubicin and their derivatives 4′-desoxydoxorubicin, 4-desmethoxydoxorubicin and 4-desmethoxydauno-

**Figure 2** Mutagenic activity of daunorubicin, doxorubicin and their derivatives on *S. typhimurium* strains. Note the different ordinate scales. Points are the mean values from 3 independent experiments: the s.e. ranged between 1 and 9.7%. Legend: A = doxorubicin; B = daunorubicin; C = 4′-desoxydoxorubicin; D = 4-desmethoxydoxorubicin; E = 4-desmethoxydaunorubicin.

rubicin was assayed on TA1535, TA1538 and their derivatives TA100 and TA98 *Salmonella typhimurium* strains. None of the compounds tested induced any revertant on the TA1535 strain, whereas they induced a significant number of revertants on TA1538. The mutagenic response was enhanced in the strains with the pKM101 plasmid, where all the compounds were mutagenic; TA98 showed a higher sensitivity than TA100 (Figure 2). The addition of S-9 mix for metabolic activation did not modify the mutagenic response of either of these strains (data not shown). The derivatives of doxorubicin and daunorubicin were more active than their respective parent compounds, except for TA98, where 4-desmethoxydaunorubicin is as mutagenic as daunorubicin.

The test of selective toxicity for DNA repair-deficient *E. coli* K12 strains showed that none of the anthracycline antibiotics has antibacterial activity on the wild-type strain AB1157, whereas an appreciable antibacterial activity was found on AB2463 (recA) and on AB2480 (recAuvrA) strains (data not shown).

On V79 Chinese hamster cells, doxorubicin and daunorubicin were found to be weakly mutagenic, whereas their derivatives showed a more marked activity, although at doses that sharply reduced the survival of the cells (Figure 3, lower part). However, when the mutagenic activity of the drugs is compared at equitoxic doses (e.g. 10% survival), only the 4′desoxydoxorubicin induced a number of mutants significantly higher than the other compounds (Figure 4).

**Figure 3** The upper part of the figure shows the cytotoxic, and the lower part the mutagenic activity of: doxorubicin (A), daunorubicin (B), 4′desoxydoxorubicin (C), 4-desmethoxydoxorubicin (D), and 4-desmethoxydaunorubicin (E) on V79 Chinese hamster cells. Points are the mean values from 3 or more independent experiments: the s.e. ranged between 0.6 and 20%.

**Figure 4** The mutagenic activity (expressed as number of 6-thioguanine-resistant clones $10^{-6}$ colony forming cells) of doxorubicin, daunorubicin and their derivatives, plotted against the surviving fraction. The regression curves are shown, and vertical bars represent the s.e. of the curves.

All the compounds were highly cytotoxic at doses varying from 0.05–0.5 $\mu g\,ml^{-1}$. 4-desmethoxydaunorubicin and 4-desmethoxydoxorubicin were the most active, since they reduced the surviving fraction by >90% at 0.25 $\mu g\,ml^{-1}$ (Figure 3, upper part). These results were obtained when the V79 cells were treated with the drugs for 1 h at 37°C; when the treatment was prolonged for 24 h, the mutagenic effect did not increase, whereas the cytotoxicity did dramatically. In fact, at doses from 0.001–0.005 $\mu g\,ml^{-1}$, no mutagenic effect could be found, and only at 0.005 $\mu g\,ml^{-1}$ was a slight increase in the number of 6-thioguanine-resistant clones observed. In contrast, doses higher than 0.01 $\mu g\,ml^{-1}$ for 24 h resulted in death of all the cells (data not shown).

Another interesting finding is that in the presence of S-9 mix, both the mutagenic and cytotoxic effects were suppressed (Table I). Although the production of inactive metabolites is consistent with this finding, the observation that the anthracycline antibiotics, after metabolic activation, bind to microsomal proteins is relevant to this point (Scheulen *et al.*, 1982).

### Discussion

As already reported for doxorubicin and daunorubicin (Seino *et al.*, 1978; Au *et al.*, 1981; Umezawa *et al.*, 1978; Benedict *et al.*, 1977), all 5 compounds were highly mutagenic in the Ames test even without S-9 mix. In contrast, on V79 Chinese hamster cells the mutagenicity: cytotoxicity ratio was low. It is therefore possible that the induced toxicity on V79 cells results at least in part from damage to cellular targets other than DNA.

Several mechanisms have been proposed to explain the biological effects of the anthracycline antibiotics. Intercalation into double helical DNA and subsequent inhibition of DNA and/or RNA synthesis have been thought to be the main molecular effect of these compounds (Zunino *et al.*, 1972; Meriwether & Bachur, 1972; Theologides *et al.*, 1968). Additional interactions with other

**Table I** Surviving fraction and mutation frequency on V79 cells of doxorubicin, daunorubicin and their derivatives with and without S-9 mix.

| | *Surviving fraction in %* | | *Mutation frequency*[b] | |
|---|---|---|---|---|
| | *−S9 mix* | *+S9 mix* | *−S9 mix* | *+S9 mix* |
| Control | 100 | 100 | 11.15 | 5.7 |
| Dimethyl-nitrosamine[a] | 100 | 94 | 10.1 | 123.3 |
| Doxorubicin[c] | 38.8 | 92.86 | 36.9 | 8.8 |
| Daunorubicin[c] | 8.15 | 87.8 | 80.3 | 2.3 |
| 4′desoxy-doxorubicin[c] | 11.1 | 97.4 | 232 | 8.1 |
| 4-desmethoxy-doxorubicin[c] | 3.9 | 98 | 187 | 2.1 |
| 4-desmethoxy-daunorubicin[c] | 2.4 | not tested | 134 | not tested |

Numbers are the mean values from at least two experiments.

[a]Dimethyl-nitrosamine 5 mM was used as positive control for the metabolic activation.

[b]Mutation frequency = 6-thioguanine-resistant clones $10^{-6}$ colony forming cells.

[c]Cells were treated with the drug at 0.25 $\mu g\,ml^{-1}$ for 1 h.

cellular components have been observed, in particular with the cell surface (Tritton & Yee, 1982; Duarte *et al.*, 1976; Scheulen, 1982). In addition, the anthracycline antibiotics can be metabolized in living cells to chemical products able to react with cellular macromolecules and can participate in oxidation-reduction reactions (Arcamone *et al.*, 1969); Sihna (1980) has shown that enzymatically activated doxorubicin and daunorubicin alkylate DNA probably via the formation of a quinone methide intermediate.

Moreover, Scheulen *et al.* (1982) have proposed that the enzymatic activation of these substances can lead to the formation of reactive intermediates that covalently bind to cellular proteins.

In contrast, the mutagenic activity of these substances in eukaryotic cells shown in this and other studies (Suter *et al.*, 1980; Marquardt *et al.*, 1976) indicates that anthracycline antibiotics introduce lesions in DNA that lead to errors during replication and/or repair. The nature of the damage and the way in which the cells deal with it can be inferred from data obtained in *S. typhimurium* with and without pKM101 plasmid and on *E. coli* K12 strains with different repair capabilities. The different responses of TA1535 and TA1538 to the five compounds suggest that the intercalation of the molecule into DNA is probably involved in the mutagenicity of anthracycline antibiotics. This does not exclude the possibility that other metabolic products formed inside the cells may interact with DNA, but only indicates that these compounds are frameshift mutagens.

The data obtained on *E. coli* K12 derivatives with different repair capabilities clearly show that the compounds interact with bacterial DNA. The higher toxicity exerted on the recA strain AB2463 suggests that a *recA*-dependent pathway is required for repair of the lesion.

These observations also apply to the doxorubicin and daunorubicin derivatives. However, it is evident from the present data that the derivatives are more cytotoxic and more mutagenic than the parent compounds, both in prokaryotic and in eukaryotic systems. These differences may be due to the differences in the physicochemical properties as a consequence of the modifications introduced in the anthracycline aglycone or in the amino sugar moiety (Di Marco *et al.*, 1978*a, b*). These results are in agreement with observations that the removal of the methoxyl group at position 4 of the aglycone causes a marked increase in cytotoxicity against HeLa cells *in vitro* (Supino *et al.*, 1977) and also an increase in antitumour potency (Di Marco *et al.*, 1977, 1978*b*).

A slightly increased effectiveness has also been reported with the 4′desoxy-derivative (Di Marco *et al.*, 1978*a*). These modifications may alter the potency of the antracycline antibiotics in several ways; in particular, the increased intracellular accumulation may play a relevant role (Di Marco *et al.*, 1978*a, b*).

The authors thank Dr. Penco from Farmitalia-Carlo Erba for supplying the samples of doxorubicin and daunorubicin derivatives. The technical assistance of Mrs Sidonia Saincich, Miss Alessandra Slobez and Mr Claudio Gamboz is also acknowledged. Work supported by CNR, Progetto Finalizzato Controllo della Crescita Neoplastica, grant N. 82.00361.96.

### References

ABBONDANDOLO, A., BONATTI, S., COLELLA, C. & 4 others. (1976). A comparative study of different experimental protocols for mutagenesis assay with 8-azaguanine resistance system in Chinese hamster cultured cells. *Mutat. Res.*, **37**, 293.

AMES, B.N., McCANN, J. & YAMASAKI, E. (1975). Methods for detecting carcinogens and mutagens with the Salmonella/Mammalian-microsome mutagenicity test. *Mutat. Res.*, **31**, 346.

ARCAMONE, F., FRANCESCHI, G., PENCO, S. *et al.* (1969). Adriamycin (14-hydroxydaunorubicin), a novel antitumour antibiotic. *Tetrahedron Lett.*, **13**, 1007.

ARCAMONE, F., PENCO, S., VIGEVANI, A. & 8 others. (1975). Synthesis and antitumor properties of new glycosides of daunomycinone and adriamycinone. *J. Med. Chem.*, **18**, 703.

AU, W.W., BUTLER, M.A., MATNEY, T.S. & LOO, T.L. (1981). Comparative structure-genotoxicity study of three amino-antraquinone drugs and doxorubicin. *Cancer Res.*, **41**, 376.

BACHMANN, B.J. (1972). Pedigree of some mutant strains of *Escherichia coli* K12. *Bacteriol. Rev.*, **36**, 525.

BENEDICT, W.F., BAKER, M.S., HAROUN, L., CHOI, E. & AMES, B.N. (1977). Mutagenicity of cancer chemotherapeutic agents in the Salmonella/microsome test. *Cancer Res.*, **37**, 2209.

BLUM, R. & CARTER, S. (1974). Adriamycin: A new anticancer drug with significant clinical activity. *Ann. Intern. Med.*, **80**, 249.

DAVIS, B. & MINGIOLI, E.S. (1950). Mutants of *Escherichia coli* requiring methionine or vitamin $B_{12}$. *J. Bacteriol.*, **60**, 17.

DI MARCO, A., GAETANI, M., DORIGOTTI, L., SOLDATI, M. & BELLINI, O. (1963). Daunomycin: A new antibiotic with antitumor activity. *Tumori*, **49**, 203.

DI MARCO, A., CASAZZA, A.M., DASDIA, T. & 6 others. (1977). Changes of activity of daunorubicin, adriamycin and stereoisomers following the introduction or removal of hydroxyl groups in the amino sugar moiety. *Chem. Biol. Interact.*, **19**, 291.

DI MARCO, A., CASAZZA, A.M., GIULIANI, F. & 5 others. (1978*a*). Synthesis and antitumor activity of 4-demethoxyadriamycin and 4-demethoxy-4′-epiadriamycin. *Cancer Treat. Rep.*, **62,** 375.

DI MARCO, A., CASAZZA, A., SORANZO, C. & PRATESI, G. (1978*b*). Effect of various substitutions in position 1, 2, 3 and 4 of 4-demethoxydaunorubicin and 4-demethoxyadriamycin. *Cancer Chemother. Pharmacol.*, **1,** 249.

DUARTE-KARIM, M., RUYSSCHAERT, J.M. & HILDEBRAND, J. (1976). Affinity of adriamycin to phospholipids: a possible explanation for cardiac mithocondrial lesions. *Biochem. Biophys. Res. Commun.*, **71,** 658.

KONOPA, J. (1983). Adriamycin and daunomycin induce interstrand DNA crosslinks in HeLa S3 cells. *Biochem. Biophys. Res. Commun.*, **110,** 819.

MARQUARDT, H., PHILIPS, F.S. & STERNBERG, S.S. (1976). Tumorigenicity *in vivo* and induction of malignant transformation and mutagenesis in cell cultures by adriamycin and daunomycin. *Cancer Res.*, **36,** 2065.

MERIWETHER, W.D. & BACHUR, N.R. (1972). Inhibition of DNA and RNA metabolism by daunorubicin and adriamycin in L1210 mouse leukemia. *Cancer Res.*, **32,** 1137.

SCHEULEN, M.E., KAPPUS, H., NIENHAUS, A. & SCHMIDT, C.G. (1982). Covalent protein binding of reactive adriamycin metabolites in rat liver and rat heart microsomes. *J. Cancer Res. Clin. Oncol.*, **103,** 39.

SEINO, Y., NAGAO, M., YAHAGI, T., HOSHI, A., KAWACHI, T. & SUGIMURA, T. (1978). Mutagenicity of several classes of antitumor agents to *Salmonella typhimurium* TA98, TA100 and TA92. *Cancer Res.*, **38,** 2148.

SINHA, B.K. (1980). Binding specificity of chemically and enzimatically activated anthracycline anticancer agents to nucleic acids. *Chem. Biol. Interact.*, **30,** 67.

SUPINO, R., NECCO, A., DASDIA, T., CASAZZA, A.M. & DI MARCO, A. (1977). Relationshops between effects on nucleic acids synthesis in cell cultures and cytotoxicity of 4-demethoxy derivatives of daunorubicin and adriamycin. *Cancer Res.*, **37,** 4523.

SUTER, W., BRENNAND, J., McMILLAN, S. & FOX, M. (1980). Relative mutagenicity of antineoplastic drugs and other alkylating agents in V79 Chinese hamster cells, independence of cytotoxic and mutagenic responses. *Mutat. Res.*, **73,** 171.

THEOLOGIDES, A., YABRO, J. & KENNEDY, B.J. (1968). Daunomycin inhibition of DNA and RNA synthesis. *Cancer*, **21,** 16.

TRITTON, T.R. & YEE, G. (1982). The anticancer agent adriamycin can be actively cytotoxic without entering the cells. *Science*, **217,** 248.

UMEZAWA, K., SAWAMURA, M., MATSUSHIMA, T. & SUGIMURA, T. (1978). Mutagenicity of aclacinomycin A and daunomycin derivatives. *Cancer Res.*, **38,** 1782.

ZUNINO, F., GAMBETTA, R., DI MARCO, A. & ZACCARA, A. (1972). Interaction of daunomycin and its derivatives with DNA. *Biochim. Biophys. Acta*, **277,** 489.